# Improved crystal orientation and physical properties from single-shot XFEL stills

**DOI:** 10.1107/S1399004714024134

**Published:** 2014-11-28

**Authors:** Nicholas K. Sauter, Johan Hattne, Aaron S. Brewster, Nathaniel Echols, Petrus H. Zwart, Paul D. Adams

**Affiliations:** a Physical Biosciences Division, Lawrence Berkeley National Laboratory, Berkeley, CA 94720, USA

**Keywords:** X-ray free-electron lasers, single-shot exposures

## Abstract

X-ray diffraction patterns from still crystals are inherently difficult to process because the crystal orientation is not uniquely determined by measuring the Bragg spot positions. Only one of the three rotational degrees of freedom is directly coupled to spot positions; the other two rotations move Bragg spots in and out of the reflecting condition but do not change the direction of the diffracted rays. This hinders the ability to recover accurate structure factors from experiments that are dependent on single-shot exposures, such as femtosecond diffract-and-destroy protocols at X-ray free-electron lasers (XFELs). Here, additional methods are introduced to optimally model the diffraction. The best orientation is obtained by requiring, for the brightest observed spots, that each reciprocal-lattice point be placed into the exact reflecting condition implied by Bragg’s law with a minimal rotation. This approach reduces the experimental uncertainties in noisy XFEL data, improving the crystallographic *R* factors and sharpening anomalous differences that are near the level of the noise.

## Abstract 2

X-ray free-electron laser crystallography relies on the collection of still-shot diffraction patterns. New methods are developed for optimal modeling of the crystals’ orientations and mosaic block properties.

## 1. Introduction   

**Figure 1:**
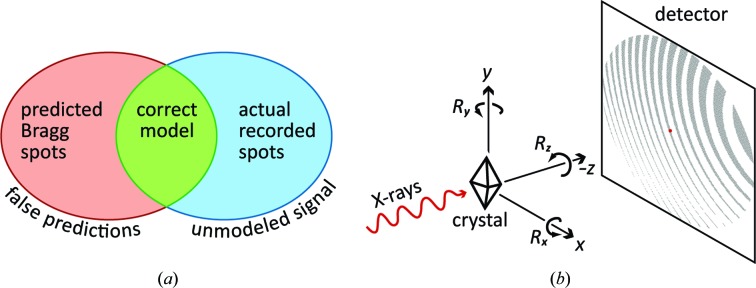
Relation between the observation and prediction of Bragg spots. (*a*) The aim of data processing is to exactly predict the Bragg spots that are actually recorded. (*b*) Definition of the laboratory coordinate system, with incident X-rays traveling in the −*z* direction and rotations *R_x_, R_y_* and *R_z_* along the three principal axes. Only the rotation *R_z_* has a direct effect on Bragg spot positions.

Recent high-resolution crystallographic structure determinations at X-ray free-electron lasers have required 10^4^–10^5^ still shots to achieve adequate signal to noise (Boutet *et al.*, 2012 ▶; Redecke *et al.*, 2013 ▶; Barends *et al.*, 2013 ▶; Liu *et al.*, 2013 ▶), thus placing severe demands on the limited amounts of available sample and instrument time. A critical question that has yet to be answered is whether systematic improvements in the way that the data are treated would lessen these requirements. The hope is that a more accurate model of the experiment will help to identify the specific pixels in the diffraction image that contain Bragg signal rather than background or noise, leading to better structure-factor estimates from fewer images. In a previous paper (Hattne *et al.*, 2014 ▶), we raised the issue of whether the shape of Bragg spots can be precisely modeled on either empirical grounds or by considering crystal mosaicity and spectral dispersion. Here, we probe a similarly fundamental issue: is the set of Bragg spots predicted by the model an exact match to the set of Bragg spots actually recorded, or is there a slight mismatch that gives either falsely predicted spots or true signals that are not modeled (Fig. 1 ▶ *a*)?

The idea of a mismatch between predicted and observed Bragg spots is a well understood consequence of having only a single still shot from which to deduce the crystal orientation. Generally speaking, the positions of the brightest Bragg spots are used by an indexing algorithm (Steller *et al.*, 1997 ▶; Sauter *et al.*, 2004 ▶) to produce an approximate orientation. Numerical optimization is then used to refine the model (Paciorek *et al.*, 1999 ▶), for example with a least-squares target function,
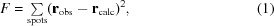
that seeks to minimize the squared-distance residual between measured spot centroid positions, **r**_obs_, and those calculated from the model, **r**_calc_. Model parameters that need to be optimized are the unit-cell lengths and angles, as well as the three orthogonal misorientation angles *R*_*x*_, *R*_*y*_ and *R_z_*. On a still shot, unfortunately, only one of these misorientation angles has an explicit effect on **r**_calc_, namely the rotation *R_z_* around the beam axis (Fig. 1 ▶ *b*) that turns both the crystal and the resulting diffraction pattern in lockstep. The orthogonal misorientations *R_x_* and *R_y_* do not change the calculated spot centroids **r**_calc_; rather, these rotations move new Bragg spots into reflecting positions. As a consequence, the intersecting set of spots that are both observed and modeled is reduced in size. Synchrotron-based experiments do not face this limitation, since the goniometer mount permits crystals to be exposed in several orientations with exactly known relationships, thus coupling all three misorientation angles to the calculated spot positions from two or more exposures (Sauter *et al.*, 2004 ▶, 2006 ▶).

To assess whether the inability to refine the *R_x_* and *R_y_* misorientation angles has practical implications for XFEL data, we measured the success rate for refining the orientations of simulated still-shot diffraction patterns for photosystem I (PSI). Test conditions represented the simplest possible case, with idealized monochromatic radiation from a constant-flux, zero-divergence source illuminating zero-mosaicity crystals with a known size and orientation. Indeed, we find that the straightforward approach of applying the target function (1) for the refinement of six unit-cell and three rotational parameters diverges from the known solution in a considerable fraction of cases (see §[Sec sec3]3). We therefore tested additional methods to produce a closer match to the true orientation.

**Figure 2:**
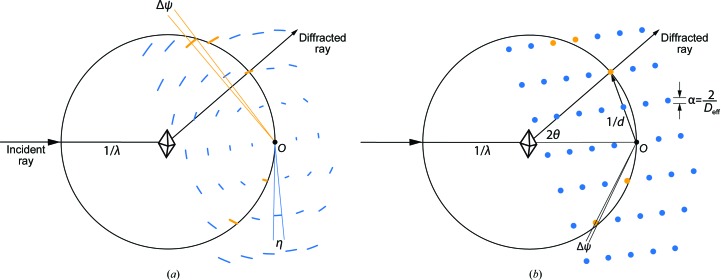
The relationship between mosaicity and mosaic block size. Ewald sphere diagram identifying the reciprocal-lattice points (gold) actually observed in the diffraction pattern. A goal of modeling is to adjust the effective mosaicity (*a*) and effective mosaic domain size (*b*) together so as to bring all the observed points into contact with the sphere of reflection of radius 1/λ (where λ is the wavelength), but not the unobserved (blue) points. (*a*) Mutual rotation of mosaic blocks spreads the points into concentric arcs (spherical caps in three dimensions) subtending a constant angle η at the reciprocal-lattice origin *O*, with η interpreted as the full-width effective mosaicity. A lattice point diffracts if its centroid (midpoint) can be brought onto the sphere of reflection with a rotation Δψ ≤ η/2. (*b*) Expansion of the reciprocal-lattice points into constant-sized spheres, reflecting the finite size of mosaic blocks (Nave, 1998 ▶) or, equivalently, the domain-size broadening (Scherrer, 1918 ▶). The sphere diameter α is inversely proportional to the effective mosaic block size *D*_eff_. The sphere size illustrated in (*b*) falls short of that needed to completely model the observed reflections.

A second problem arising with still shots is that model centroids do not exactly meet the reflecting conditions to infinite precision (Fig. 2 ▶); instead, we assume that the experiment has some imperfections allowing Bragg spots to be observed slightly off-condition. For synchrotron experiments this has been successfully modeled as a parameter describing the effective mosaicity (Winkler *et al.*, 1979 ▶; Rossmann *et al.*, 1979 ▶; Bolotovsky & Coppens, 1997 ▶), a composite parameter that encompasses the effects of beam divergence, mutual rotation of mosaic blocks (illustrated in Fig. 2 ▶ *a*) and block-to-block differences in unit-cell parameters. These effects scale in direct proportion to the diffraction angle (Nave, 1998 ▶; Juers *et al.*, 2007 ▶) and are thus useful for modeling the high-resolution reflections (Fig. 2 ▶ *a*). However, they account for vanishingly few Bragg spots in the low-resolution limit. In our experience with XFEL still shots taken at the CXI instrument at LCLS (Kern *et al.*, 2012 ▶, 2013 ▶; Hattne *et al.*, 2014 ▶), we observe numerous low-angle spots that cannot be modeled by effective mosaicity. Specifically, if we increase the mosaicity value to predict all the low-resolution spots that are actually observed, then the model predicts far too many high-resolution spots. This problem can be solved by complementing the model with a term describing the mosaic block size (Fig. 2 ▶ *b*; Nave, 1998 ▶, 2014 ▶; Juers *et al.*, 2007 ▶; Battye *et al.*, 2011 ▶). We investigate here how to optimally adjust these two effects so as to model both the high-resolution and low-resolution reflections.

In the present study, we make the approximation of treating diffraction as arising from monochromatic X-rays (see §[Sec sec4]4), as this provides a reasonable starting point for still images.

## 2. Methods   

### 2.1. Additional restraints for orientational refinement   

**Figure 3:**
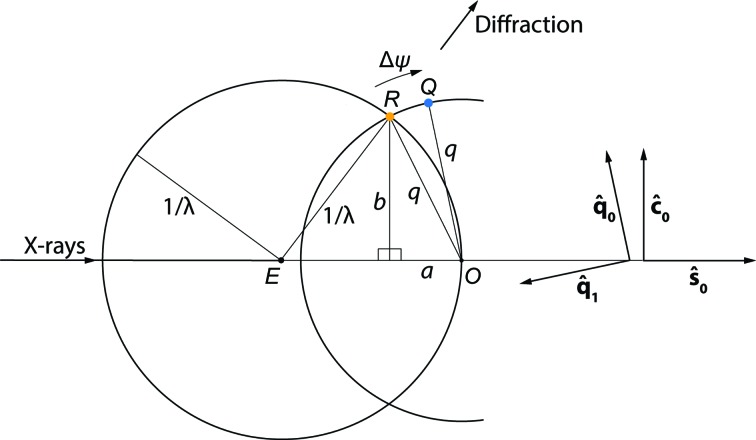
The construction of Δψ. As stated in the text, the sign of the rotation Δψ bringing *Q* onto the Ewald sphere is considered to be negative if *Q* is outside the sphere (as shown) or positive if it is inside.

To prevent divergence while numerically optimizing the crystal orientation from still shots, we have followed the example of other authors (Jones *et al.*, 1977 ▶, Kabsch, 2014 ▶) by introducing an additional restraint that keeps model spots as close to the diffracting condition as possible (Fig. 2 ▶). For each observed Bragg spot, we define Δψ_calc_ as the magnitude of the rotation that most directly brings the modeled spot centroid from an approximate to an exact diffraction condition (Fig. 3 ▶). The model is then optimized using the new least-squares minimization target
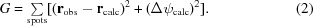
In the hybrid target (2), **r**_calc_ has a direct dependence on *R_z_*, while Δψ_calc_ depends on *R_x_* and *R_y_*; therefore, all three misorientation angles can be properly optimized. It is important to note the distinction between Δψ_calc_ and the similar angle Δϕ used in synchrotron experiments, which represents the difference in goniometer rotation angle ϕ between the observed and modeled spot centroids. The still shots discussed here do not employ a goniometer spindle, so instead of bringing the reciprocal-lattice point into a reflecting condition by an angular rotation Δϕ around a physical spindle, we simply construct a rotation axis (different for each Bragg spot; Fig. 3 ▶) that brings the model centroid into the reflection condition with the smallest possible angle Δψ_calc_.

For (2) we evaluate **r**_obs_ − **r**_calc_ in units of millimetres and Δψ_calc_ in units of radians/(2π). Thus, both terms are weighted roughly equally (within an order of magnitude) and both are numerically on a convenient scale (below 1) for Gauss–Newton nonlinear least-squares minimization as implemented within the *Computational Crystallography Toolbox* (*cctbx*; Grosse-Kunstleve *et al.*, 2002 ▶). We note that other authors have used relative weighting schemes using inverse-variance factors (Kabsch, 2014 ▶).

To find the optimal model, the target expression (2) is recast in terms of fundamental experimental quantities including the beam direction 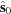, the wavelength λ, the crystal orientation and the unit-cell parameters. The parameter dependence of the **r**_obs_ − **r**_calc_ term has been described elsewhere (Paciorek *et al.*, 1999 ▶); here, we focus on the quantity Δψ_calc_(**h**) that corresponds to a reciprocal-lattice point with Miller index **h** (hereafter referred to as Δψ). The reflection **h** arises from a crystal with reciprocal-space orientation matrix **A** as defined previously (Rossmann *et al.*, 1979 ▶), 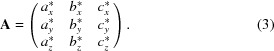
The matrix elements of (3) are the projections of the reciprocal-space unit-cell vectors a*, b* and c* onto the laboratory axes *x*, *y* and *z*. As we use a vectorial approach it is not strictly important how the orthonormal laboratory axes are chosen, but Fig. 1 ▶(*b*) gives one possible convention. The reciprocal-space coordinates (laboratory frame) of the reflection are
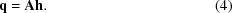
The paradigm for calculating Δψ is shown in Fig. 3 ▶, depicting reciprocal space with origin *O*, the Ewald sphere of radius 1/λ centered at *E* and the reciprocal-lattice point *R* on the Ewald sphere surface meeting the reflecting conditions described by Bragg’s law, giving rise to the diffracted ray 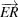 = 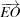 + 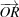, or **s**_1_ = **s**_0_ + **r** in conventional notation. However, the current model for the lattice point (4) predicts not the position *R* (= **r**) but a position *Q* (= **q**) that is slightly off the Ewald sphere. The angle Δψ is defined as the rotation needed to bring point *Q* onto *R* and thus into the exact diffracting condition. This rotation is around a unit vector 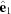 perpendicular to plane *EOQ* and pointing into the page. We find it useful to define Δψ as a signed quantity: negative if *Q* is outside the Ewald sphere (as shown) and positive if it is inside the sphere.

By first defining 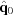 as the unit-length vector along **q**,
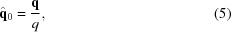
we can then define the orthonormal vectors
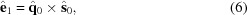
and
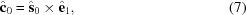
which allows us to write a vector expression for *R*, 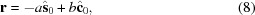
with positive quantities *a* and *b* obtained by solving the right triangles of Fig. 3 ▶:
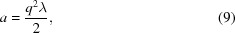


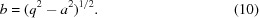
The desired angle Δψ between **q** and **r** can now be calculated *via* the tan^−1^() function. As an aid for visualizing this, we define the orthonormal vectors 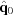 and
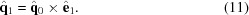
We then express Δψ in terms of the projection of **r** onto the opposite and adjacent legs of a right triangle, 
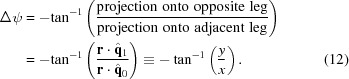
Finally, we determine the optimal model of the experiment by minimizing (2) over the set of bright observed reflections. We use iterative nonlinear least-squares methods, requiring the evaluation of the first derivatives of Δψ with respect to a set of underlying parameters {*p*} (Appendix *A* 1). All of the experimental quantities 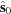, λ and **A** may be considered to be functions of one or more underlying parameters, for example the unit vector 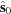 has two directional degrees of freedom corresponding to its latitudinal and longitudinal intersection with the Ewald sphere, and the underlying parameter of λ is λ itself. Furthermore, the orientation matrix **A** is a function of three Euler angles, as well as three unit-cell lengths and three unit-cell angles with appropriate constraints for crystal symmetry (Sauter *et al.*, 2006 ▶). Alternatively, the **A** matrix could be parameterized in terms of the *R_x_* and *R_y_* misorientations (Fig. 1 ▶ *b*). Details concerning appropriate parameterizations will be described elsewhere.

### 2.2. Best-fit crystal properties for the prediction of model spots   

Fig. 2 ▶ depicts the familiar Ewald-sphere construction that is useful for visualizing which reciprocal-lattice points are near the reflecting condition implied by Bragg’s law. To gain a realistic prediction of which spots are observed, we do not require lattice points to be precisely on the sphere; rather, we accept points that are close to the sphere, within a certain tolerance.

Fig. 2 ▶(*a*) portrays the usual tolerance criterion attributed to mosaicity, requiring that spot *i* can be brought onto the sphere by a minimal rotation through angle Δψ_*i*_ about the origin, such that
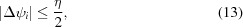
where the angle *η* is interpreted as the effective mosaicity. We use ‘effective’ to emphasize the limitation that we are not distinguishing among the numerous underlying physical phenomena that produce a spread of Δψ_*i*_ values consistent with (13), such as mutual rotation of mosaic blocks, block-to-block variation in unit-cell parameters and beam divergence. Instead, we group together all factors that produce a resolution-independent angular spread into the η parameter.

In contrast, Fig. 2 ▶(*b*) illustrates an alternative model with all reciprocal-lattice points being assigned the same reciprocal-space diameter α, leading to observed diffraction when
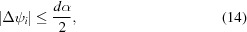
dependent on the resolution *d*. A basic result from far-field diffraction theory is that the size of the reciprocal-space spot is inversely proportional to the size of the coherently diffracting object. For a one-dimensional crystal of length *D* placed normal to the beam, the diffracted spot width is α = 2/*D*, while for three-dimensional solids an additional geometrical factor arises from the Fourier transform of the crystal shape. For mosaic crystals, the spot size is determined by the average shape transform of the mosaic blocks. We will ignore these details here, and simply state that
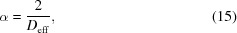
where the effective size *D*_eff_ accounts for the fact that coherently scattering mosaic blocks may occur in the crystal with a distribution of shapes and sizes.

In real still-shot experiments with monochromatic light, we expect the Δψ_*i*_ values for observed spots to have a distribution that reflects both resolution-independent (13) and resolution-dependent (14) effects. To optimize our experimental model, we therefore seek to find parameters η and α that form the minimal envelope
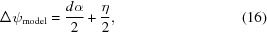
that accounts for all the observations
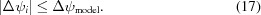
We constructed plots of Δψ *versus* resolution for the brightest spots (see §[Sec sec3]3), and evaluated two curve-fitting techniques to determine the best η and α values for predicting the full set of lattice points (both bright and weak reflections) that intersect the Ewald sphere.

#### 2.2.1. Analytical least-squares curve-fitting for η and α   

In this approach, the bright-spot data are grouped into resolution bins. For each bin we evaluate which observation gives the largest magnitude of Δψ_*i*_. We assign this value (|Δψ|_max_) to represent the envelope of observations at the average resolution *d* of that bin. The immediate goal is to use linear least-squares methods to derive the best curve Δψ_model_(*d*) to fit the |Δψ|_max_. It is worth noting that once the maximum magnitude is selected for each resolution bin, the full spread of observations is no longer used. We constructed a resolution bin for every 25 bright spots; thus, only 1/25 of the Δψ_*i*_ values are actually used for least-squares fitting.

The function to be minimized is
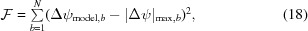
where the sum is over all resolution bins *b*. With (16), this becomes
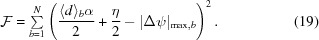
Minimizing this expression (Appendix *B*) gives the best least-squares estimates for η and α.

#### 2.2.2. Maximum-likelihood formalism for estimating η and α   

A drawback of the least-squares approach, as noted, is that it selects only the bright observations with extreme values of Δψ_*i*_ from which to derive the limiting envelope Δψ_model_ (16). Here, we develop an alternative approach that uses all the data together, which consistently gives smaller and more realistic values for the half-width mosaicity (see §[Sec sec3]3).

We start with the premise of choosing a model envelope with the greatest posterior probability (McCoy, 2004 ▶),
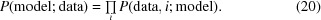
Inspired by Bayes’ theorem, this formulation posits that the posterior probability of the model, given the data, is the product over all Bragg spots *i* of the likelihood of the data, given the model.

**Figure 4:**
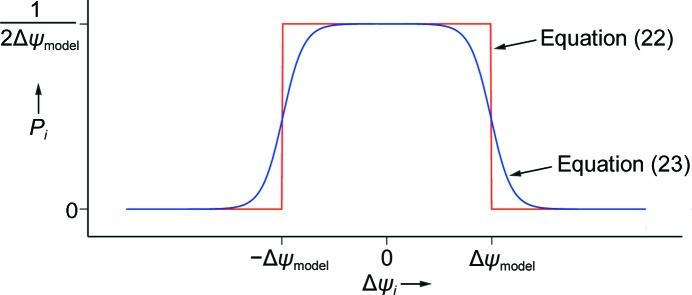
Probability of the observation Δψ_*i*_ given the model Δψ_model_.

What is the likelihood *P*(data, *i*; model) of observing the angular offset Δψ_*i*_ given the model? According to the paradigm of (17), there is 100% likelihood that
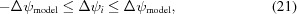
or, stated in other terms, the likelihood is a top-hat function (Fig. 4 ▶), 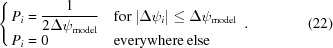
It is clear that there is an optimal solution in which the Δψ_model_ envelope (see §[Sec sec3]3) is just large enough to include the observations. If |Δψ_model_| is too small, some observations will fall outside the envelope and the probability of the data *P_i_* will be zero. Conversely, if |Δψ_model_| is too large, the probability (22) again approaches zero asymptotically. A potential problem is that the top-hat function (22) is not continuous and cannot be differentiated at the boundaries Δψ_model_, so it is not suitable for iterative parameter-optimization techniques. We therefore modify the equation to include sigmoidal functions *f* and *g* that smoothly model the step-up and step-down discontinuities in the top-hat, respectively,
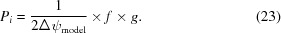



Suitable expressions for *f* and *g* may be derived from the logistic functional form (1 + *e* ^−*x*^)^−1^, 
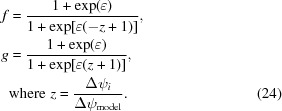
Here, the parameter ∊ controls the steepness of the sigmoid. We choose a constant value of ∊ = 10 throughout (Fig. 4 ▶), giving a fairly gently slope; values larger than 50 would give steep top-hat sides.

As Fig. 4 ▶ shows, expression (23) preserves the overall width and height of the top-hat function, but is everywhere differentiable, allowing us to proceed with parameter optimization (Appendix *B*).

### 2.3. Data-processing workflow   

**Figure 5:**
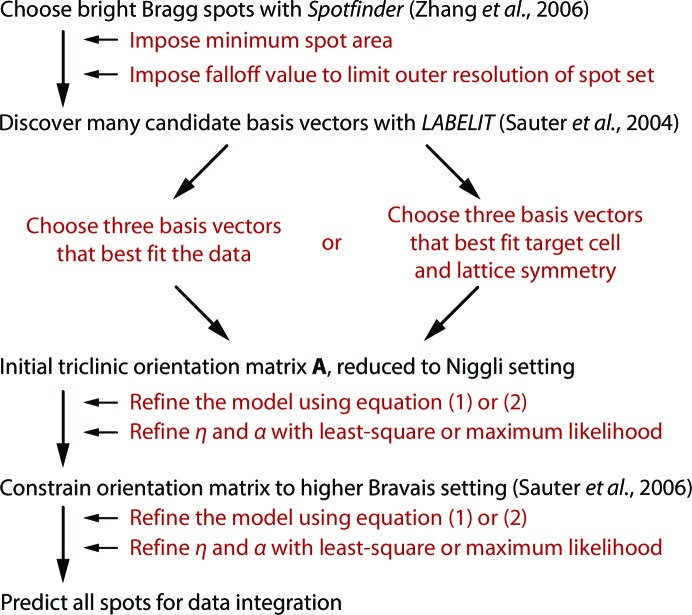
*cctbx.xfel* data-processing workflow. Steps leading to integrated data are listed in black, while choices that are under user control are listed in red. Program parameters controlling these choices are given in a tutorial at http://cci.lbl.gov/xfel.

The new procedures of §§2.1 and 2.2 were incorporated into the program *cctbx.xfel* (Hattne *et al.*, 2014 ▶). All modeling of still diffraction images was implemented within a data-processing workflow (Fig. 5 ▶) that relies exclusively on the centroid positions of bright candidate Bragg spots identified by a spotfinding procedure (Zhang *et al.*, 2006 ▶). Weak spots, spot shapes and spot intensities are not treated here, although they will be included in future work, and we make the additional approximation that the incident X-rays are mono­chromatic. Three candidate basis vectors from the program *LABELIT* (Sauter *et al.*, 2004 ▶) are chosen to span the lattice formed by the bright spots, thus forming an initial triclinic model (Steller *et al.*, 1997 ▶). After refinement of this model with either target function (1) or (2), the model is constrained to the appropriate Bravais symmetry (Sauter *et al.*, 2006 ▶) and re-refined against either target (1) or (2). Integrated data from multiple images were merged with the *cxi.merge* component of *cctbx.xfel* as described in Hattne *et al.* (2014 ▶). Intensity statistics were analyzed with *phenix.xtriage* (Zwart *et al.*, 2005 ▶) and structural models were refined with *phenix.refine* (Adams *et al.*, 2010 ▶). Tutorials on the operation of *cctbx.xfel* are given at http://cci.lbl.gov/xfel.

### 2.4. Analysis of simulated diffraction data   

Simulated still-shot diffraction patterns from PSI were obtained from James Holton (LBNL) and are available at http://bl831a.als.lbl.gov/example_data_sets/Illuin/LCLS. The images were created with the program *fastBragg* as described in Kirian *et al.* (2010 ▶, 2011 ▶), utilizing modeled structure factors from Protein Data Bank entry 1jb0. Spatially coherent simulations of randomly oriented parallelepiped nanocrystals (17 × 17 × 30 unit cells; cell lengths *a* = *b* = 281, *c* = 165.2 Å) were performed, assuming constant-flux, polarized, monochromatic radiation (λ = 1.32 Å) with zero divergence impinging on a pixel-array detector with pixel size (0.11 mm)^2^ at a distance of 129 mm from the sample. Solvent scattering and shot noise were added so as to effectively limit the resolution to about 3.3 Å. At very low resolutions (*d* > 60 Å) the simulation exhibits diffraction fringes between Bragg spots as previously observed for PSI (Chapman *et al.*, 2011 ▶; not shown); however, the present paper attempts to analyze only the central Bragg peak, and we limit our analysis to the 15–3.5 Å resolution range. Angular misorientation between the *cctbx.xfel* models and the true crystal orientations used for the simulation were calculated after accounting for the orientational ambiguities owing to the lattice symmetry operators (sixfold along *z* and twofold along *xy*).

### 2.5. Application to experimental XFEL data   

Thermolysin diffraction patterns were reprocessed from a previously described 2.1 Å resolution data set (Hattne *et al.*, 2014 ▶) that is publicly archived at the Coherent X-ray Imaging Data Bank (accession ID 23). The typical crystal size was approximately 2 × 3 × 1 µm (Sierra *et al.*, 2012 ▶). Since the thermolysin structure contains a single Zn atom, it was possible to use the signal-to-noise ratio of the anomalous difference electron density as a metric for the quality of data processing. We therefore limited the analysis to data (runs 16–27) collected at a wavelength of 1.269 Å, which is slightly more energetic than the Zn *K* edge at 1.284 Å. As this discarded runs 71–73 that included the highest resolution data, we were obliged to choose a slightly lower diffraction cutoff (2.2 Å) than that previously reported. We selected 14 041 images containing >15 Bragg spots for further processing using either the same protocol employed in the previous analysis (Hattne *et al.*, 2014 ▶; column ‘NM’ in Table 2 ▶) or the new procedures of §§2.1 and 2.2. Diffraction from up to two separate crystal lattices was analyzed for each image.

## 3. Results   

To assess how well data-processing algorithms can model still-shot crystal orientations and structure factors, we began by analyzing simulated diffraction images, reasoning that this would provide a comparison against the known true values. Aggregate results for six different protocols are presented in Table 1 ▶. We next evaluated processing performance on actual XFEL data from the protease thermolysin, with the results given in Table 2 ▶.

### 3.1. Judging the model accuracy based on experimentally accessible measures   

**Figure 6:**
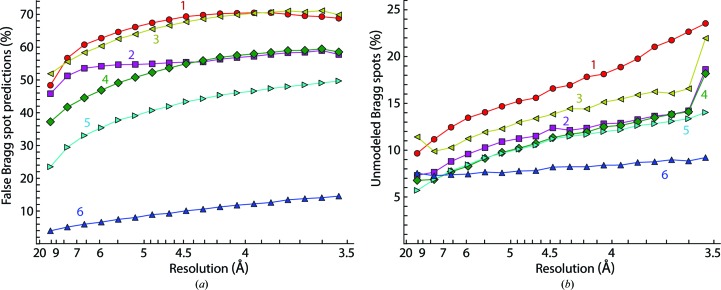
Agreement of model and data. ‘True knowledge’ of the system used for the PSI simulation gives the fraction of Bragg spots falsely predicted (*a*) and the fraction of Bragg spots in the simulation that remain unmodeled (*b*) for each of the six protocols listed in Table 1 ▶. Integrated data from all simulated images are grouped into reciprocal-space shells of equal volume ranging from 15 to 3.5 Å.

For the development of data-processing algorithms, simulated data confer the unique advantage of knowing the ‘true’ hidden variables used to generate the simulation. For each of the six protocols used to model the simulated PSI data (Table 1 ▶), we can therefore calculate what fraction of Bragg spots are falsely predicted by the model and what fraction of Bragg spot signal in the simulated images remain un­modeled (Table 1 ▶ and Fig. 6 ▶); the results ranged from poor (protocols 1 and 3) to very good (protocol 6). Unexpectedly, we found that some data-quality measures that would normally be accessible in a real experiment offered only limited insight into the true model quality. For example, one might expect that protocols producing poor models might also have a reduced success rate in indexing the lattice, yet we find instead that the poorest protocols still index ≥94% of the images. Combined with the fact that with a realistically heterogeneous distribution of crystals it would be difficult to precisely count the total number of ‘hits’ that contain Bragg spots, we must conclude that the overall count of integrated and merged images offers little insight into the model quality.

Two other measures, the best-fit effective mosaicity and the number of negative measurements, could potentially be useful for understanding model quality (Table 1 ▶). Protocols 1 and 3, which produce the most misoriented models and the largest fractions of falsely predicted Bragg spots, also yield the highest model mosaicities. This is consistent with the idea that a misoriented model places the reciprocal-lattice centers of the observed spots far from the Ewald sphere (high Δψ_*i*_), requiring large mosaicity values (Fig. 2 ▶ *a*) to bring the centroids back into diffracting position. Smaller average mosaicities over the whole population of images, as for protocol 6, are therefore an indication of a better-conforming model. In a similar fashion, the number of negative measurements (Table 1 ▶) partly reflects the prevalence of falsely predicted Bragg spots that give ‘signals’ containing Gaussian noise, with positive and negative measurements evenly distributed around zero. Once again, protocol 6, with the best-conforming models, also generates the lowest percentage of negative measurements. The multiplicity of observation (Tables 1 ▶ and 2 ▶), or the average number of repeat measurements of the same Miller index, is inversely related to the model quality: more accurate models give lower multiplicity. While this may be counterintuitive, it is a direct consequence of smaller, more well conforming effective mosaicity values predicting fewer spots, while at the same time a greater fraction of the predicted spots have true signal.

Other data-quality metrics, which rely on an analysis of data after they are scaled and merged, certainly reflect the model quality, but their interpretation is complicated by other factors. *I*/σ(*I*), which is maximal in the best protocols (Tables 1 ▶ and 2 ▶), not only reflects the modeling of individual images but for real still shots is influenced by the protocols chosen to scale and merge the images (Hattne *et al.*, 2014 ▶), by non-isomorphism among crystals, by other shot-to-shot differences in beam and sample, and by the partial nature of the structure-factor measurements from still images (not treated here). Finally, the *L* and *N*(*Z*) statistical tests of structure-factor quality that are widely used in other contexts to detect twinning (Padilla & Yeates, 2003 ▶) are also usefully correlated with the model accuracy (Table 1 ▶), but are subject to the same caveats as discussed for *I*/σ(*I*).

### 3.2. Accuracy depends on optimal spotfinding and indexing parameters   

Fig. 5 ▶ indicates the decision points that we investigated in our data-processing workflow. The first two relate to the spotfinding practices used to obtain the set of candidate Bragg spots for indexing. We found it necessary to carefully customize the program parameters (Zhang *et al.*, 2006 ▶) for individual data sets. For the PSI simulated data, the largest and best set of candidate spots was obtained by lowering the minimum spot area to one pixel; comparing protocols 4 and 1 in Table 1 ▶ shows that the model quality is degraded by imposing a stricter minimum spot area of two pixels, giving a smaller set of Bragg spots from which to index. For the thermolysin data (and indeed for most real XFEL data sets) we were obliged to use a minimum spot area of two pixels, since the more aggressive limit of one pixel produces too many candidate spots that represent noise, thereby degrading the indexing result. Secondly, for both PSI and thermolysin the candidate Bragg spot set was extended to the highest resolution by lowering the ‘method 2 cutoff’ (Zhang *et al.*, 2006 ▶) to 5%. The more stringent cutoff of 20% used by default for rotation data sets in *LABELIT* eliminates too many actual high-resolution candidate spots required for an optimal indexing solution (compare protocols 4 and 2). We optimize both spotfinding parameters in practice by visualizing their effects within a graphical interface.

A third decision point reflects the method for choosing basis vectors to form the unit cell; the quality of the orientation matrix was markedly improved by providing target values for the unit-cell lengths and angles as previously described (Hattne *et al.*, 2014 ▶); compare protocols 4 and 3.

### 3.3. Best accuracy and best signal are achieved with the hybrid target function   

Beyond these factors, we found that the inclusion of a Δψ term in the orientational refinement (2) greatly improves the model angular orientation, producing mosaicity values that conform better to the experiment, smaller sets of unwanted ‘negative measurements’ and more acceptable merged structure factors as evaluated by *R*_iso_ (protocols 5 and 6, Tables 1 ▶ and 2 ▶). The use of (2) also improves the *L* and *N*(*Z*) statistical tests noted above, which are often used to detect phenomena such as twinning (Padilla & Yeates, 2003 ▶), but which for us simply give a general measure of structure-factor quality (Tables 1 ▶ and 2 ▶). We observe the best results (protocol 6) when (2) is applied sequentially to both refinement steps executed by *cctbx.xfel*: the initial triclinic refinement that independently modifies six unit-cell dimensions (three lengths and three angles) and three orientational degrees of freedom, as well as a second refinement step during which Bravais symmetry constraints are applied. Failure to apply the orientational Δψ term during either of these steps allows the model to diverge (protocols 4 and 5 and data not shown).

Following all of the best practices (protocol 6) for simulated PSI data (Table 1 ▶) leads to a high fraction (>99%) of orientational models being within 0.1° of the correct alignment, produces an average mosaicity identical to the true value of 0.0° and models the average domain block size with a value (5100 Å) very close to the true value of 4780–4950 Å for a 17 × 17 × 30 unit cell crystallite.

For the thermolysin XFEL data, protocol 6 also leads to the lowest crystallographic *R* factors (*R*_work_ and *R*_free_ of 20.6 and 26.0%, respectively, at 2.2 Å resolution; Table 2 ▶) when automatically refining the structure using the published structure 4ow3 as input. Protocol 5, which uses refinement target (1) for the second cell-refinement step, produces much poorer *R* factors (about four percentage points higher). Furthermore, the improvements conveyed by protocol 6 also allow us to clearly identify the anomalous difference signal from natively bound Zn^2+^ in a Fourier map at a level of 5.9 standard deviations (σ) above the noise (Table 2 ▶), as opposed to 3.0σ for protocol 5. For a weak anomalous signal such as this, the improvement owing to the orientational Δψ term therefore makes a crucial difference in unambiguously identifying a metal site.

### 3.4. Physical properties of the crystals   

**Figure 7:**
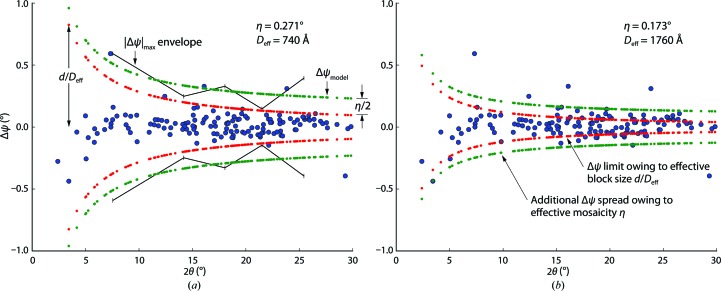
Two methods for fitting the mosaicity and mosaic block size. Δψ values for bright spots from a single thermolysin still image (blue circles) are plotted as a function of the diffraction angle 2θ, which is inversely related to the resolution *d* by Bragg’s law. A wider spread of Δψ values is observed at low 2θ. The best integrated intensities are obtained by finding the function Δψ_model_ (green curve) that minimally envelopes the spots. (16) breaks Δψ_model_ into a resolution-dependent term containing the effective mosaic block size *D*_eff_ (inner red curve) and a peripheral zone of constant width determined by the effective mosaic spread η. Alternate algorithms determine these parameters either by (*a*) least-squares fit of the |Δψ|_max_ values determined for resolution bins or (*b*) maximum-likelihood treatment of all of the data. Approach (*b*) consistently gives more realistic fits with smaller η and larger *D*_eff_ values. Plots reflect refinement results from protocol 6 (Table 2 ▶).

Once the crystal orientation has been refined as above, the residual values of Δψ clearly show the mosaic structure of crystals when plotted against the diffraction angle (Fig. 7 ▶). The average block sizes of the mosaic domain *D*_eff_ are reflected in the wide spread of Δψ residuals observed at low resolution (14), while the narrow taper at high resolution is a measure of the effective mosaicity angle η (13). Indeed, it is critical to derive correct values for these parameters when modeling an image; an overall envelope Δψ_model_ that is too narrow will fail to include real Bragg spot signals, while an overly wide envelope will falsely predict Bragg spots, thus mixing Gaussian noise into the average structure factors. Of the two methods evaluated for determining η and *D*_eff_, the maximum-likelihood approach (Fig. 7 ▶ *b*) consistently outperformed the least-squares method (Fig. 7 ▶ *a*) and was ultimately adopted for all of the data presented in Tables 1 ▶ and 2 ▶. This judgment was based on lower η for the simulated PSI data set (which ideally should be 0°), a lower percentage of negative measurements for both data sets, better structure-factor quality tests, better crystallographic *R* factors for the thermolysin structure refinement and higher significance levels for the Zn^2+^ anomalous peak (data not shown).

## 4. Discussion   

This paper describes methods for correctly predicting the set of Bragg spots observed in diffraction still shots. Previous indexing approaches (Kirian *et al.*, 2010 ▶) modeled the orientation of simulated PSI crystals to an r.m.s. error of 0.06°. Here, we reduce the r.m.s. misorientation to 0.038° by introducing an additional term in the least-squares refinement target function (2), and quantify the extent to which better-oriented models have a superior ability to predict the actual set of Bragg spots in the data (Fig. 6 ▶). We show that improvements of this scale lead to more accurate structure factors and enhance the ability to detect anomalous (Bijvoet) differences. Optimal models for extracting structure factors will make XFEL experiments more practical: a recent SAD phasing study using Gd-derivatized lysozyme required ∼60 000 still shots to obtain adequate signal to noise (Barends *et al.*, 2013 ▶), but for many proteins it is challenging to prepare this many crystals, and XFEL beam time is scarce. Better still-shot treatment will also facilitate those synchrotron experiments for which high radiation sensitivity precludes more than one shot per crystal (Grimes *et al.*, 1998 ▶).

Traditional modeling of rotation data sets (Kabsch, 2010 ▶) includes an effective mosaicity parameter that captures the effects of beam divergence, as well as differences in unit-cell parameters and orientation among mosaic blocks within the crystal. The mosaicity value controls the number density of Bragg spots predicted by the model, and is thus crucial for correctly modeling rotation data and still-shot data alike. However, for still-shot data we find that mosaicity by itself is insufficient, and a second parameter must be introduced to properly model the resolution-dependency of the observed density of Bragg spots. At the lowest resolutions (small diffracting angles) more diffraction spots are observed when the average block size of mosaic domains is small. This additional parameter, which can be determined by analyzing Δψ_max_ (the largest angular rotation needed to bring model spot centroids into ideal Bragg diffracting conditions), is crucial for modeling still shots from both simulated data and real experimental data from XFEL sources. We included the domain block size parameter in our recent analyses of photosystem II (Kern *et al.*, 2013 ▶, 2014 ▶) and thermolysin (Hattne *et al.*, 2014 ▶; protocol ‘NM’ in Table S2), although the data-treatment method (Fig. 7 ▶) is presented here for the first time.

These methods improve the correspondence between the set of spots observed and those predicted by the model. An important issue that must still be resolved is how to relate the intensities measured from still shots to those derived from rotation exposures, which have the benefit of fully moving each reciprocal-lattice point through the reflection condition. Still shots clearly lead to a partial measurement of the Bragg spot since the intensity is only sampled at one point of the rocking curve. We propose that the Δψ concept offers a framework to approach this partiality problem: with all other things equal (crystal size, incident beam intensity, unit-cell parameters) the intensity of the partial measurement reaches a peak at |Δψ| = 0 and falls off to zero at large |Δψ|. This information may be sufficient to determine the relative scaling between duplicate measurements of a Bragg spot from numerous crystals, although the details of the scaling procedure have yet to be worked out.

We reiterate that the formulae presented in this paper rest on the assumption that the incident radiation is monochromatic, allowing us to represent the reflection condition (Fig. 2 ▶) with an Ewald sphere of clearly defined radius 1/λ. This is a very good approximation for synchrotron sources that can typically be tuned to very small bandpasses (10^–4^). Indeed, recently reported data collected with still shots (Axford *et al.*, 2014 ▶) could likely benefit from the improved model accuracies achieved here. Also, recent synchrotron techniques that scan rapidly through numerous crystals by loop-based rastering (Gati *et al.*, 2014 ▶), capillary flow (Stellato *et al.*, 2014 ▶), acoustic injection (Roessler *et al.*, 2013 ▶) or microfluidic sample delivery (Heymann *et al.*, 2014 ▶) could benefit from accurate processing techniques that enable still-shot data collection. Fast synchrotron-source pseudo-stills offer tremendous potential for avoiding radiation damage (Owen *et al.*, 2014 ▶) while probing biologically relevant conformational details that can only be detected at room temperature (Keedy *et al.*, 2014 ▶). The situation with XFEL sources is more complicated, since the stochastic lasing process generates hard X-ray bandpasses on the order of 0.5% (Emma *et al.*, 2010 ▶). The monochromatic model is a useful starting point for XFEL data analysis (Table 2 ▶), which we are currently working to extend to explicitly model finite-width X-ray spectra. Additionally, recent self-seeding techniques (Amann *et al.*, 2012 ▶) offer the possibility of future XFEL data collection with a narrow-bandpass incident spectrum.

## Supplementary Material

Appendices A and B. DOI: 10.1107/S1399004714024134/wa5077sup1.pdf

## Figures and Tables

**Table 1:** Processing outcome on simulated PSI data with different protocols

	Non-optimal spotfinding and indexing			Equation (1) only	Equations (1) and (2)	Best practice†
Protocol	1	2	3	4	5	6
Refinement target						
Initial triclinic cell	Equation (1)	Equation (1)	Equation (1)	Equation (1)	Equation (2)	Equation (2)
Constrained hexagonal cell	Equation (1)	Equation (1)	Equation (1)	Equation (1)	Equation (1)	Equation (2)
Indexing practices						
Spotfinder spot area (pixels)	2	1	1	1	1	1
Spotfinder method 2 cutoff (%)	5	20	5	5	5	5
Target unit cell	Provided	Provided	Not given	Provided	Provided	Provided
Indexing results						
Total No. of images	20000	20000	20000	20000	20000	20000
No. of integrated and merged images	19706	19490	18926	19608	19998	19984
Model accuracy						
R.m.s. *R_z_* misorientation ()	0.039	0.041	0.040	0.017	0.017	0.017
R.m.s. *R_x_* + *R_y_* misorientation ()	0.379	0.586	1.108	0.584	0.083	0.031
R.m.s. total angular misorientation ()	0.381	0.588	1.109	0.584	0.085	0.035
Median total angular misorientation ()	0.130	0.087	0.134	0.078	0.054	0.021
No. of outliers >0.1 misoriented	13085	9264	13136	7971	4566	172
False Bragg predictions, 153.5 (%)	65.0	54.3	64.7	51.6	40.0	9.1
Unmodeled Bragg spots, 153.5 (%)	15.4	10.9	13.3	10.4	10.0	8.0
Half-width mosaicity‡ () (true value 0)	0.101	0.080	0.098	0.050	0.025	0.000
Mosaic block size‡ () (true value 4850)	5160	4660	4960	4660	4780	5100
Integrated data results						
Individual image CC (%)	51.6	54.1	50.6	56.3	61.2	70.1
No. of measurements, 153.5	38398465	35264088	38755029	36948917	32210606	22817281
Positive measurements, 153.5	26322687	25323211	26836439	26779348	24458975	19303707
Negative measurements (%)	31	28	31	28	24	15
Structure-factor merging						
Unique Miller indices, 153.5	92204	92204	92204	92204	92204	92204
Multiplicity of observation	286	275	291	290	265	209
Completeness (%)	100	100	100	100	100	100
*I*/(*I*)	33.4	36.0	33.8	38.0	40.9	46.0
CC_iso_ *versus* 1jb0 (based on intensities) (%)	96.6	96.6	95.8	96.8	97.5	99.0
*R*_iso_ *versus* 1jb0 (based on intensities) (%)	36.7	33.0	35.5	32.1	27.7	18.1
Structure-factor quality tests						
|*L*| (acentric theoretical = 0.5)	0.270	0.299	0.282	0.301	0.320	0.358
*L* ^2^ (acentric theoretical = 0.333)	0.109	0.131	0.118	0.132	0.148	0.182
*P*(*Z*) maximum deviation (acentric)	0.263	0.223	0.245	0.222	0.197	0.154
*P*(*Z*) maximum deviation (centric)	0.364	0.332	0.341	0.330	0.306	0.243

† *cctbx.xfel* now runs protocol 6 by default, while the other protocols may be accessed by changing the program parameters described at http://cci.lbl.gov/xfel.

‡ Half-width mosaicity and mosaic block size were fitted by the maximum-likelihood approach outlined in Appendix *B*. The values reported here are *D*_eff_ and 1/, respectively, where is the average over all merged images.

**Table 2:** Processing outcome on measured XFEL still shots from thermolysin

	Previous work†	Equation (1) only	Equations (1) and (2)	Best practice
Protocol	NM	4	5	6
Refinement target				
Initial triclinic cell	Equation (1)	Equation (1)	Equation (2)	Equation (2)
Constrained hexagonal cell	Equation (2)	Equation (1)	Equation (1)	Equation (2)
Fitting of mosaicity and block size	Least squares	Maximum likelihood	Maximum likelihood	Maximum likelihood
Indexing results‡				
Total No. of hits with >15 Bragg spots	14041	14041	14041	14041
No. of integrated and merged lattices	11151	12097	11958	12551
Model accuracy				
Half-width mosaicity ()	0.471	0.292	0.286	0.168
Mosaic block size ()	2920	4320	4320	4220
Integrated data results				
Individual image CC (%)	32.5	32.0	32.3	40.2
No. of measurements, 512.2	5793963	6605566	6538120	5036076
Positive measurements, 512.2	3893827	4297065	4265829	3626262
Negative measurements (%)	33	35	35	28
Structure-factor merging				
Unique Miller indices, 512.2	17156	17198	17193	17297
Multiplicity of observation	222	245	243	207
Completeness (%)	97.9	98.2	98.2	98.8
*I*/(*I*)	41.1	36.1	36.7	56.7
CC_iso_ *versus* 4ow3 (based on intensities) (%)	90.1	86.8	86.6	94.7
*R*_iso_ *versus* 4ow3 (based on intensities) (%)	22.5	23.6	23.4	18.0
Structure-factor quality tests				
|*L*| (acentric theoretical = 0.5)	0.340	0.302	0.304	0.376
*L* ^2^ (acentric theoretical = 0.333)	0.169	0.137	0.138	0.202
*P*(*Z*) maximum deviation (acentric)	0.159	0.201	0.196	0.121
*P*(*Z)* maximum deviation (centric)	0.238	0.271	0.265	0.198
Quality of refined structure				
*R*_work_ (%)	21.9	24.5	24.2	20.6%
*R*_free_ (%)	27.9	29.6	29.8	26.0
Zn^2+^ anomalous difference map peak height ()	3.5	2.9	3.0	5.9

† This column replicates the method in our previous publication (Hattne *et al.*, 2014 ▶) used to derive the thermolysin structure (PDB entry 4ow3).

‡ For the thermolysin data analysis, candidate Bragg spots were chosen with a minimum spot area of two square pixels.

## Footnotes

1 Supporting information has been deposited in the IUCr electronic archive (Reference: WA5077).
