# Supplementary material for: Improved crystal orientation and physical properties from single-shot XFEL stills
Source: Acta Crystallogr D Biol Crystallogr. 2014 Nov 28;70(Pt 12):3299–309. doi: 10.1107/S1399004714024134 (PMC4257623; doi:10.1107/S1399004714024134)
Supplement: Supplementary file 1 [file d-70-03299-sup1.pdf]

BIOLOGICAL
CRYSTALLOGRAPHY

Volume 70 (2014)

Supporting information for article:

Improved crystal orientation and physical properties from single-shot XFEL stills

Nicholas K. Sauter, Johan Hattne, Aaron S. Brewster, Nathaniel Echols, Petrus H. Zwart and Paul D. Adams

Appendix A. Partial derivatives of $\Delta\psi$.

Here, we present partial derivatives of $\Delta\psi$ with respect to the experimental quantities (beam direction, wavelength, and crystal orientation matrix), necessary for parameter optimization with target expression (2).

A.1 Partial derivatives with respect to $\hat{\mathbf{s}}_\mathbf{0}$

For the data presented in Table 1 the direction of the incident beam is exactly known ($\hat{\mathbf{s}}_\mathbf{0} = -\hat{z}$). However, it may be necessary to refine the $\hat{\mathbf{s}}_\mathbf{0}$ model for some experimental situations, so the method is presented here. We assume that $\hat{\mathbf{s}}_\mathbf{0}$ is expressed as a function of parameters p_i with known partial derivatives $\partial\hat{\mathbf{s}}_\mathbf{0}/\partial p_i$. Then based on equations (4) to (12) we have:

$$\frac{\partial \mathbf{q}}{\partial p_i} = 0, \tag{A.1}$$

$$\frac{\partial \hat{\mathbf{q}}_\mathbf{0}}{\partial p_i} = 0, \tag{A.2}$$

$$\frac{\partial \hat{\mathbf{e}}_\mathbf{1}}{\partial p_i} = \hat{\mathbf{q}}_\mathbf{0} \times \frac{\partial \hat{\mathbf{s}}_\mathbf{0}}{\partial p_i}, \tag{A.3}$$

$$\frac{\partial \hat{\mathbf{c}}_\mathbf{0}}{\partial p_i} = \frac{\partial \hat{\mathbf{s}}_\mathbf{0}}{\partial p_i} \times \hat{\mathbf{e}}_\mathbf{1} + \hat{\mathbf{s}}_\mathbf{0} \times \frac{\partial \hat{\mathbf{e}}_\mathbf{1}}{\partial p_i}, \tag{A.4}$$

$$\frac{\partial a}{\partial p_i} = \frac{\partial b}{\partial p_i} = 0, \tag{A.5}$$

$$\frac{\partial \mathbf{r}}{\partial p_i} = -a\frac{\partial \hat{\mathbf{s}}_\mathbf{0}}{\partial p_i} + b\frac{\partial \hat{\mathbf{c}}_\mathbf{0}}{\partial p_i}, \tag{A.6}$$

$$\frac{\partial \hat{\mathbf{q}}_\mathbf{1}}{\partial p_i} = \hat{\mathbf{q}}_\mathbf{0} \times \frac{\partial \hat{\mathbf{e}}_\mathbf{1}}{\partial p_i}. \tag{A.7}$$

Defining $y = \mathbf{r} \cdot \hat{\mathbf{q}}_\mathbf{1}$ and $x = \mathbf{r} \cdot \hat{\mathbf{q}}_\mathbf{0}$ we have:

$$\frac{\partial y}{\partial p_i} = \frac{\partial \mathbf{r}}{\partial p_i} \cdot \hat{\mathbf{q}}_\mathbf{1} + \mathbf{r} \cdot \frac{\partial \hat{\mathbf{q}}_\mathbf{1}}{\partial p_i}, \tag{A.8}$$

$$\frac{\partial x}{\partial p_i} = \frac{\partial \mathbf{r}}{\partial p_i} \cdot \hat{\mathbf{q}}_\mathbf{0}, \tag{A.9}$$

and finally

$$\frac{\partial \Delta\psi}{\partial p_i} = \frac{x\dfrac{\partial y}{\partial p_i} - y\dfrac{\partial x}{\partial p_i}}{x^2 + y^2}. \tag{A.10}$$

A.2 Partial derivatives with respect to λ

In the work presented here the wavelength λ is exactly known; however, it may be important to refine the wavlength in XFEL experiments where the incident radiation is from a self-amplified pulse, and is therefore different for each shot. If we wish to refine λ directly as a parameter, then

$$\frac{\partial \mathbf{q}}{\partial \lambda} = \frac{\partial \hat{\mathbf{q}}_\mathbf{0}}{\partial \lambda} = \frac{\partial \hat{\mathbf{e}}_\mathbf{1}}{\partial \lambda} = \frac{\partial \hat{\mathbf{c}}_\mathbf{0}}{\partial \lambda} = 0, \tag{A.11}$$

$$\frac{\partial a}{\partial \lambda} = \frac{q^2}{2}, \tag{A.12}$$

$$\frac{\partial b}{\partial \lambda} = \frac{-a}{b}\frac{q^2}{2}, \tag{A.13}$$

$$\frac{\partial \mathbf{r}}{\partial \lambda} = -\hat{\mathbf{s}}_\mathbf{0}\frac{\partial a}{\partial \lambda} + \hat{\mathbf{c}}_\mathbf{0}\frac{\partial b}{\partial \lambda}, \tag{A.14}$$

$$\frac{\partial \hat{\mathbf{q}}_\mathbf{1}}{\partial \lambda} = 0, \tag{A.15}$$

$$\frac{\partial y}{\partial \lambda} = \frac{\partial \mathbf{r}}{\partial \lambda} \cdot \hat{\mathbf{q}}_\mathbf{1}, \tag{A.16}$$

$$\frac{\partial x}{\partial \lambda} = \frac{\partial \mathbf{r}}{\partial \lambda} \cdot \hat{\mathbf{q}}_\mathbf{0}, \tag{A.17}$$

and finally

$$\frac{\partial \Delta\psi}{\partial \lambda} = \frac{x\frac{\partial y}{\partial \lambda} - y\frac{\partial x}{\partial \lambda}}{x^2 + y^2}. \tag{A.18}$$

A.3 Partial derivatives with respect to $\mathbf{A}$

Assuming that the reciprocal space orientation matrix $\mathbf{A}$ is expressed as a function of parameters p_j with known partial derivatives $\partial \mathbf{A}/\partial p_j$, we have:

$$\frac{\partial \mathbf{q}}{\partial p_j} = \frac{\partial \mathbf{A}}{\partial p_j}\mathbf{h}, \tag{A.19}$$

$$\frac{\partial}{\partial p_j}(\mathbf{q} \cdot \mathbf{q}) = \frac{\partial}{\partial p_j} q^2 = 2\mathbf{q} \cdot \frac{\partial \mathbf{q}}{\partial p_j} \quad \text{and} \quad \frac{\partial q}{\partial p_j} = \frac{\mathbf{q} \cdot \frac{\partial \mathbf{q}}{\partial p_j}}{q}, \tag{A.20}$$

$$\frac{\partial \hat{\mathbf{q}}_\mathbf{0}}{\partial p_j} = \frac{q\frac{\partial \mathbf{q}}{\partial p_j} - [\mathbf{q} \cdot \frac{\partial \mathbf{q}}{\partial p_j}]\hat{\mathbf{q}}_\mathbf{0}}{q^2}, \tag{A.21}$$

$$\frac{\partial \hat{\mathbf{e}}_\mathbf{1}}{\partial p_j} = \frac{\partial \hat{\mathbf{q}}_\mathbf{0}}{\partial p_j} \times \hat{\mathbf{s}}_\mathbf{0}, \tag{A.22}$$

$$\frac{\partial \hat{\mathbf{c}}_{\mathbf{0}}}{\partial p_j} = \hat{\mathbf{s}}_{\mathbf{0}} \times \frac{\partial \hat{\mathbf{e}}_{\mathbf{1}}}{\partial p_j}, \tag{A.23}$$

$$\frac{\partial a}{\partial p_j} = \lambda \frac{\partial \mathbf{q}}{\partial p_j} \cdot \mathbf{q}, \tag{A.24}$$

$$\frac{\partial b}{\partial p_j} = \frac{2 - q^2\lambda^2}{2b} \frac{\partial \mathbf{q}}{\partial p_j} \cdot \mathbf{q}, \tag{A.25}$$

$$\frac{\partial \mathbf{r}}{\partial p_j} = -\frac{\partial a}{\partial p_j}\hat{\mathbf{s}}_{\mathbf{0}} + \frac{\partial b}{\partial p_j}\hat{\mathbf{c}}_{\mathbf{0}} + b\frac{\partial \hat{\mathbf{c}}_{\mathbf{0}}}{\partial p_j}, \tag{A.26}$$

$$\frac{\partial \hat{\mathbf{q}}_{\mathbf{1}}}{\partial p_j} = \frac{\partial \hat{\mathbf{q}}_{\mathbf{0}}}{\partial p_j} \times \hat{\mathbf{e}}_{\mathbf{1}} + \hat{\mathbf{q}}_{\mathbf{0}} \times \frac{\partial \hat{\mathbf{e}}_{\mathbf{1}}}{\partial p_j}, \tag{A.27}$$

$$\frac{\partial y}{\partial p_j} = \frac{\partial \mathbf{r}}{\partial p_j} \cdot \hat{\mathbf{q}}_{\mathbf{1}} + \mathbf{r} \cdot \frac{\partial \hat{\mathbf{q}}_{\mathbf{1}}}{\partial p_j}, \tag{A.28}$$

$$\frac{\partial x}{\partial p_j} = \frac{\partial \mathbf{r}}{\partial p_j} \cdot \hat{\mathbf{q}}_{\mathbf{0}} + \mathbf{r} \cdot \frac{\partial \hat{\mathbf{q}}_{\mathbf{0}}}{\partial p_j}, \tag{A.29}$$

and finally

$$\frac{\partial \Delta\psi}{\partial p_j} = \frac{x\dfrac{\partial y}{\partial p_j} - y\dfrac{\partial x}{\partial p_j}}{x^2 + y^2}. \tag{A.30}$$

For parameters p_j that represent pure rotations of the crystal, we have the simplification that $\frac{\partial \mathbf{q}}{\partial p_j} \cdot \mathbf{q} = 0$ in equations (A.20), (A.21), (A.24) and (A.25).

Appendix B. Best fit mosaicity η and block size D_{eff}.

Here we present two procedures to compute the optimal effective full-width mosaicity η and spot width α.

B.1 Analytical least-squares expression

Optimal parameters α and η are obtained when the partial derivatives of (19) are zero:

$$\frac{\partial \mathcal{F}}{\partial \alpha} = \frac{\partial \mathcal{F}}{\partial \eta} = 0. \tag{B.1}$$

This leads to a system of normal equations

$$\alpha \sum_{b=1}^{N} \frac{\langle d \rangle_b^2}{2} + \eta \sum_{b=1}^{N} \frac{\langle d \rangle_b}{2} = \sum_{b=1}^{N} \langle d \rangle_b |\Delta\psi|_{\text{max},b} \tag{B.2}$$

$$\alpha \sum_{b=1}^{N} \frac{\langle d \rangle_b}{2} + \eta \frac{N}{2} = \sum_{b=1}^{N} |\Delta\psi|_{\text{max},b}, \tag{B.3}$$

that can be written in matrix form and directly solved:

$$\begin{pmatrix} \sum_{b=1}^{N} \frac{\langle d\rangle_b^2}{2} & \sum_{b=1}^{N} \frac{\langle d\rangle_b}{2} \\ \sum_{b=1}^{N} \frac{\langle d\rangle_b}{2} & \frac{N}{2} \end{pmatrix} \begin{pmatrix} \alpha \\ \eta \end{pmatrix} = \begin{pmatrix} \sum_{b=1}^{N} \langle d\rangle_b |\Delta\psi|_{\mathsf{max},b} \\ \sum_{b=1}^{N} |\Delta\psi|_{\mathsf{max},b} \end{pmatrix}. \tag{B.4}$$

B.2 Maximum likelihood formalism

The obvious parameters to be optimized are α and η. However, we wish to enforce positivity constraints to keep the values physically meaningful. In order to avoid special methods to impose parameter bounds, we define the free parameters as the logarithms:

$$\xi_\alpha = \ln(\alpha) \quad \text{and} \quad \xi_\eta = \ln(\eta). \tag{B.5}$$

The requisite partial derivatives are

$$\frac{\partial \Delta\psi_{\mathsf{model}}}{\partial \xi_\alpha} = \frac{d_i \alpha}{2} \quad \text{and} \quad \frac{\partial \Delta\psi_{\mathsf{model}}}{\partial \xi_\eta} = \frac{\eta}{2}. \tag{B.6}$$

For either parameter ξ, and with z as defined in (24),

$$\frac{\partial z}{\partial \xi} = \frac{\Delta\psi_i}{(\Delta\psi_{\mathsf{model}})^2} \frac{\partial \Delta\psi_{\mathsf{model}}}{\partial \xi}, \tag{B.7}$$

$$\frac{\partial f}{\partial \xi} = \epsilon \times f \times \frac{\exp(\epsilon(-z+1))}{1+\exp(\epsilon(-z+1))} \frac{\partial z}{\partial \xi}, \tag{B.8}$$

$$\frac{\partial g}{\partial \xi} = -\epsilon \times g \times \frac{\exp(\epsilon(z+1))}{1+\exp(\epsilon(z+1))} \frac{\partial z}{\partial \xi}, \tag{B.9}$$

$$\frac{\partial (fg)}{\partial \xi} = f \frac{\partial g}{\partial \xi} + g \frac{\partial f}{\partial \xi}, \tag{B.10}$$

$$\frac{\partial P_i}{\partial \xi} = \frac{1}{2} \frac{\frac{\partial (fg)}{\partial \xi} \Delta\psi_{\mathsf{model}} - fg \frac{\partial \Delta\psi_{\mathsf{model}}}{\partial \xi}}{(\Delta\psi_{\mathsf{model}})^2}. \tag{B.11}$$

Now returning to the full posterior probability expression (20), we can determine the parameters α and η by minimizing the target function

$$\mathcal{G} = -\sum_{i=1}^{\mathsf{Nobs}} \ln P_i, \tag{B.12}$$

where the sum is over all observations i. For iterative parameter optimization we utilize the partial derivatives

$$\frac{\partial \mathcal{G}}{\partial \xi} = -\sum_{i=1}^{\mathsf{Nobs}} \frac{1}{P_i} \frac{\partial P_i}{\partial \xi}. \tag{B.13}$$

A few practical details bear on the of implementation of this procedure. Care must be taken to avoid misindexing; a single Bragg spot assigned the wrong Miller index may be taken to have an erroneously large $\Delta\psi_i$ magnitude, biasing the mosaicity and domain size estimates. Conversely, parameters that produce a

too-large $\Delta\psi_{\text{model}}$ envelope predict too many Bragg spots, leading to spot overlap and misindexing. Therefore, when initial values are assigned for maximum likelihood refinement (by using the analytical least-squares expression) we insist that the estimated domain size exceeds some minimum value. We choose a physically reasonable minimum of 10 unit cells for the edge of a domain ($D_{\text{eff}} \geq 10 \times \sqrt[3]{\text{unit cell volume}}$).

Another concern is that the exponential expressions in (24), (B.8), and (B.9) are susceptible to arithmetic overflow if the argument is too large; the arguments must therefore be tested prior to each exponential execution.
